# Membranous CD24 expression as detected by the monoclonal antibody SWA11 is a prognostic marker in non-small cell lung cancer patients

**DOI:** 10.1186/s12907-015-0019-z

**Published:** 2015-11-16

**Authors:** Michael Majores, Anne Schindler, Angela Fuchs, Johannes Stein, Lukas Heukamp, Peter Altevogt, Glen Kristiansen

**Affiliations:** Institute of Pathology, University of Bonn, Sigmund-Freud-Str. 25, D-53127 Bonn, Germany; New Pathology, Cologne, Germany; Skin Cancer Unit, German Cancer Research Center (DKFZ), Heidelberg, Germany; Department of Dermatology, Venereology and Allergology University Medical Center Mannheim, Ruprecht-Karl University of Heidelberg, Mannheim, Germany

**Keywords:** Non-small cell lung cancer, NSCLC, CD24, Immunohistochemistry, Prognostic marker

## Abstract

**Background:**

Lung cancer is one of the most common malignant neoplasms worldwide and has a high mortality rate. To enable individualized therapy regimens, a better understanding of the molecular tumor biology has still to be elucidated. The expression of the cell surface protein CD24 has already been claimed to be associated with shorter patient survival in non-small cell lung cancer (NSCLC), however, the prognostic value and applicability of CD24 immunostaining in paraffin embedded tissue specimens has been questioned due to the recent acknowledgement of restricted epitope specificity of the commonly used antibody SN3b.

**Methods:**

A cohort of 137 primary NSCLC cases was immunostained with a novel CD24 antibody (clone SWA11), which specifically recognizes the CD24 protein core and the resulting expression data were compared with expression profiles based on the monoclonal antibody SN3b. Furthermore, expression data were correlated to clinico-pathological parameters. Univariate and multivariate survival analyses were conducted with Kaplan Meier estimates and Cox regression, respectively.

**Results:**

CD24 positivity was found in 34 % resp. 21 % (SN3b) of NSCLC with a membranous and/or cytoplasmic staining pattern. Kaplan-Meier analyses revealed that membranous, but not cytoplasmic CD24 expression (clone SWA11) was associated with lympho-nodular spread and shorter overall survival times (both *p* < 0.05). CD24 expression established by SN3b antibodies did not reveal significant clinicopathological correlations with overall survival, neither for cytoplasmic nor membranous CD24 staining.

**Conclusions:**

Membranous CD24 immunoreactivity, as detected with antibody clone SWA11 may serve as a prognostic factor for lymphonodular spread and poorer overall survival. Furthermore, these results corroborate the importance of a careful distinction between membranous and cytoplasmic localisation, if CD24 is to be considered as a potential prognostic biomarker.

## Background

Lung cancer is a major cause of carcinoma related death, being responsible for 17.8 % of all cancer deaths and accounting for more than a million deaths worldwide per year [1]. Despite intense studies to improve therapy options, its prognosis has remained poor with a 5-year overall survival rate of less than 15 % [2].

In the past decade, the largest subgroup of lung cancer, i.e. non-small cell lung cancer (NSCLC), has been subjected to exerted research for a better understanding of the underlying molecular biology of lung cancer. More than ten years ago, CD24 has already been suggested as a novel and promising biomarker for carcinoma progression in NSCLC [3] and several groups have confirmed this finding on protein and transcript level [2, 4]. CD24 is a highly glycosylated protein, that binds to the cell surface through a GPI (glycosyl-phosphatidylinositol)-anchor and functions as a cell adhesion molecule and is involved in cell-cell-interaction via its P-selectin binding site [5]. CD24 has been found to be expressed by pre-B-lymphocytes [5]. It is assumed that CD24-positive cells can attach more easily to platelets and activated endothelial cells [6, 7]. Notably, CD24 has also been observed in many human carcinomas, such as ovarian cancer, renal cell cancer, breast cancer and NSCLC [3, 8–12]. In epithelial ovarian cancer high scores of cytoplasmic CD24 were highly predictive of shorter patient survival times (mean 97.8 vs. 36.5 months), whereas membranous CD24 expression seemed to have no influence on survival times. Interestingly, CD24 positivity (membranous or cytoplasmic) of prostate cancer samples was significantly associated to younger patient age and higher pT stages and a higher 3-year prostate-specific antigen (PSA) relapse rate compared with CD24-negative tumours.

In patients with gallbladder carcinoma, tumors with up-regulation of CD24 revealed lymph node metastasis and lymphovascular invasion more frequently. Moreover, up-regulation of CD24 tended to show deeper invasion depth and higher TNM stage [13]. Together, these findings support CD24 as a prognostic marker for carcinoma progression and poorer survival.

Despite these intriguing findings, major concerns regarding a lack of epitope specificity of the commonly used monoclonal antibody SN3b have been raised [14]. Recent findings indicate that the mAb (monoclonal antibody) SN3b does not bind to the protein core itself, but binds to a glycan structure that decorates the CD24 molecule. On the one hand, this motif is not present on all forms of CD24 and—on the other hand—it can be present in other epitopes irrespective of CD24 [14]. These limitations underline the need for more specific CD24 antibodies, such as the mAb SWA11 antibody that has been suggested to be more specific as it binds to the protein core [14].

As CD24 is a promising biomarker for the risk assessment of disease progression, the goal of the present study was to investigate CD24 expression in NSCLC using the novel, more specific monoclonal antibody (mAb) SWA11. Special emphasis was put on the comparison of SN3b- and SWA11-mediated CD24 detection regarding a) the subcellular distribution of CD24 expression (i.e. membranous versus cytoplasmic expression) and b) its correlation with various clinicopathological features including patient survival times.

## Methods

### Patient characteristics/ tumor samples

A cohort of 137 primary NSCLC patients, who had undergone surgery between 1995 and 2009 and who were all diagnosed in the Institute of Pathology, University of Bonn, was compiled. Tumor samples were available as formalin-fixed, paraffin-embedded tissue. According to the current WHO classification the NSCLC were classified as adenocarcinoma (AC) (*n* = 102) or squamous cell carcinoma (SCC) (*n* = 35). The male:female ratio (5:2) and mean age at diagnosis (64y; SD +/− 9y; range 24–86y) in our cohort was in accordance with the published epidemiologic distribution [1] (Table 1). No neoadjuvant radiotherapy or chemotherapy were applied before surgery. All cases were subjected to a central review based on the current WHO guidelines [1].

**Table 1:** Clinicopathological characteristics of the NSCLC cohort

		AC	SCC
		N (%)	N (%)
Tumour stage (pT)			
	1	29 (21.2 %)	5 (3.6)
	2	51 (37.2 %)	23 (16.8 %)
	3	6 (4.4 %)	6 (4.4 %)
	4	1 (0.7 %)	0 (0 %)
Nodal Status (pN)	0	37 (27.0 %)	15 (10.9 %)
	1	15 (10.9 %)	9 (6.6 %)
	2	14 (10.2 %)	3 (2.2 %)
	3	1 (0.7 %)	0 (0.0 %)
Grading (G)	1	5 (3.6 %)	0 (0.0 %)
	2	41 (29.9 %)	16 (11.6 %)
	3	44 (32.1 %)	17 (12.4 %)
Mean age at surgery		64,2	64,56
(median age)		(65)	(67)
Sex (m:w)		68:34	30:5
Median OS (months)		52	24
(SD; 95 % CI [months])		(±23.7; 5.5– 98.5)	(± 12.8;0.0– 49.0)

*SD* standard deviation; *CI* confidence interval; *n* number of cases; *OS* overall survival

### Ethics statement

This study was accomplished under the consent of the independent ethics committee of the University of Bonn (approval number 188/14).

### Tissue microarray (TMA) assembly

For construction of the tissue microarrays, suitable areas for extraction of tissue were selected and marked on haematoxylin-eosin (HE) specimen slides. A senior pathologist conducted the microscopic selection of suitable areas. The selected areas were then punched out of the corresponding paraffin donor block and inserted into the recipient block. The tissue arrayer (LD 120 Sm5-x) was purchased from Alphalys, Paris, France. All punch diameters were 0.8 mm (corresponding to an spot area of 0.79 mm^2^). Each case was represented by 3 tumor samples and 1 peritumoral non-neoplastic tissue sample.

### Immunohistochemistry

Formalin-fixed TMA sections were freshly cut (2–3 μm) and mounted on superfrost specimen slides (Thermo Fisher). Next, dewaxing was carried out with xylene and the tissue sections were gradually rehydrated. Antigen retrieval was achieved by pressure cooking in the autoclave at pH6 and under hyperfrequency wave of 360 W at 125 °C for 8 min. MAb SWA11 was diluted 1:100 and SN3b was diluted 1:50, each using a modul buffer from Medac (TA-250-PM). The immunohistochemical reaction was visualized using the detection system C-DPVB 500 HRP by Medac (all procedures were conducted according to the instructions of the manufacturer).

Positive controls, consisting of tissue samples with known positivity for the antibody, and negative controls (i.e. reactions lacking the primary antibody), were performed in parallel for each TMA slide. Expression intensity was examined in a semiquantitative manner (score 0: no staining, score 1: weak, score 2: moderate and score 3: strong staining). For statistical analyses, cases with moderate to strong expression were bundled in a ‘high expression’ and cases with negative or weak expression in a ‘low expression’ group.

### Follow-up analyses

Follow-up data were available in 93 cases. Patients suffered from primary malignant tumors of the lung and were subjected to surgical resection or diagnostic sampling between 1995 and 2009 Cases with sufficient availability of formalin-fixed, paraffin-embedded tissue entered the cohort. Survival data were obtained by the analysis of surgical and oncological medical reports as well as written request for data of the local registration offices. The survival time was defined as the time period between the date of surgery and the date of death resp. the date of the documentation as “still alive” at the last available time point. The median survival of AC cases was 52 months (SD 23.7; 95 % CI: 5.5–98.5; *n* = 67), compared to SCC with 24 months (SD 12; 95 % CI: 0–49.0; *n* = 26). 62 patients died within the follow-up period. The remaining 31 patients were documentes as “still alive” at the last available time point. Only in 13 patients without documentation of death the follow-up period was less than 60 months.

### Statistical analysis

For statistical analysis the SPSS software v. 21.0 was used. For evaluation of the correlation between expression of CD24 and clinicopathological parameters Fisher’s exact test was used. Univariate survival analysis included Kaplan-Meier-analyses with log-rank testing for the estimation of differences in survival times. For multivariate survival analysis, the Cox regression model was used. All cutoff values of significance were set *p* < 0.05 with two-sided testing.

## Results

### Immunohistochemical detection of CD24 expression using clone SWA11 and SN3b

**Fig 1:**
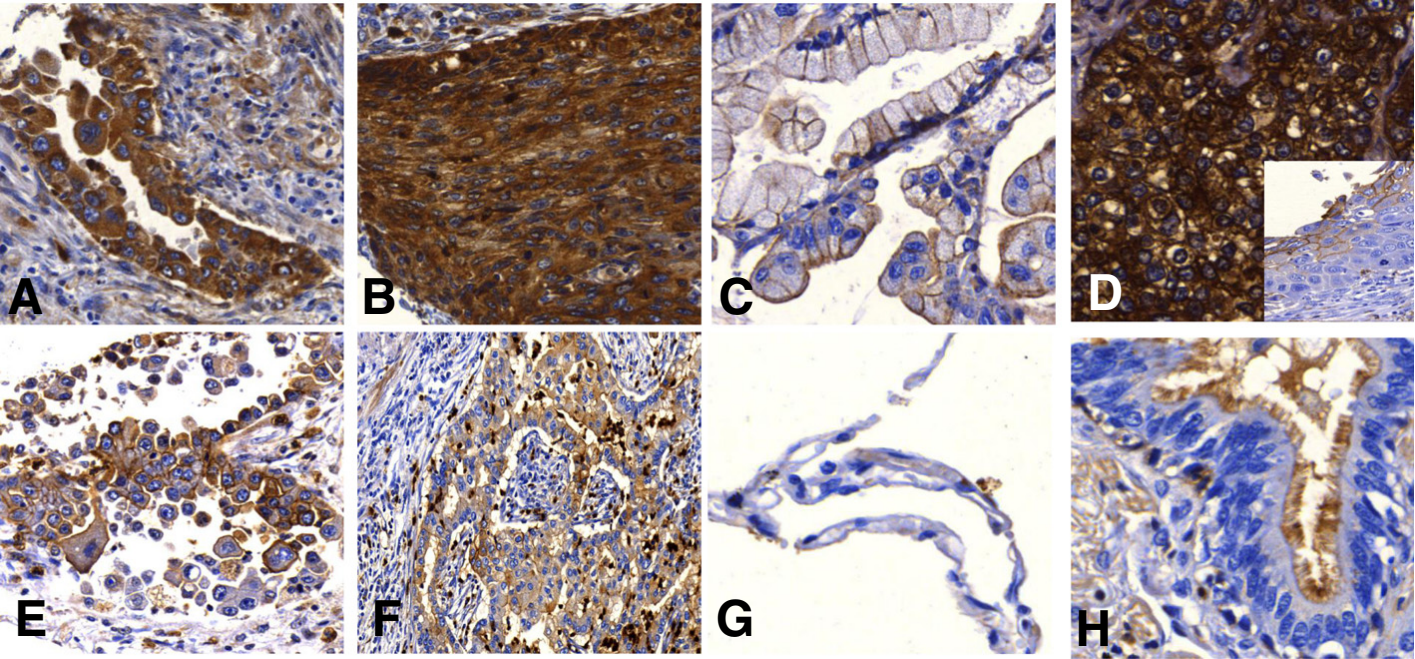
The immunohistochemical characterization reveals membranous and/or cytoplasmic CD24 (mAb SWA11) expression. Strong cytoplasmic CD24 expression is found in a proportion of both AC (**a**) and SCC (**b**, **d**) specimens. Membranous CD24 expression can be pronounced with only scant or even absent cytoplasmic staining as shown in the AC (**c**). Also, both membranous and cytoplasmic CD24 detection can be found in some instances (**d**), the insert is showing the corresponding squamous carcinoma in-situ with membranous staining. Simultaneous membranous and cytoplasmic CD24 expression is also found in AC specimens (**e**, **f**). In normal tissue, alveolar epithelial cells do not express CD24 (**g**), whereas CD24 staining is found at the apical cell membrane of bronchial respiratory epithelia (**h**)

Using the mAb SWA11, 47 of 137 (34.3 %) NSCLC revealed CD24 expression (either cytoplasmic or membranous) (Table 2). CD24 expression was observed more frequently in adenocarcinomas (AC) than in squamous cell carcinomas (SCC). In AC cytoplasmic expression was observed more frequently than membranous expression. In SCC, both cyptoplasmic and membranous expression was rare. Normal lung parenchyma (i.e. alveolar surface cells) showed no expression of CD24. Bronchial epithelium showed a strong membranous and cytoplasmic staining of the brush border (Fig. 1).

**Table 2:** Cytoplasmic and membranous expression of CD24

*SWA11 (mAb clone)*			*SN3b (mAB clone)*		
	AC	SCC		AC	SCC
Cytoplasmic	N (%)	N (%)	Cytoplasmic	N (%)	N (%)
0	45 (32.6 %)	19 (13.8 %)	0	76 (55.1 %)	31 (22.5 %)
1	22 (15.9 %)	8 (5.8 %)	1	12 (8.7 %)	1 (0.7 %)
2	17 (12.3 %)	4 (2.9 %)	2	7 (5.1 %)	2 (1.4 %)
3	18 (13.0 %)	4 (2.9 %)	3	1 (0.7 %)	0 (0 %)
	AC	SCC		AC	SCC
Membranous	N (%)	N (%)	Membranous	N (%)	N (%)
0	68 (49.3 %)	21 (15.2 %)	0	64 (46.4 %)	30 (21.7 %)
1	21 (15.2 %)	5 (3.6 %)	1	10 (7.2 %)	2 (1.4 %)
2	8 (5.8 %)	4 (2.9 %)	2	12 (8.7 %)	2 1.4 %)
3	5 (3.6 %)	5 (3.6 %)	3	10 (7.2 %)	0 (0 %)

Staining intensities are determined as follows:

0: negative or equivocal, 1: weak, 2: moderate and 3: strong CD24 staining

*Using* the mAb *SN3b,* 29 of 137 (21.2 %) NSCLC revealed CD24 expression (either cytoplasmic or membranous) (Table 2). As above, CD24 expression was observed more frequently in adenocarcinomas (AC) than in squamous cell carcinomas (SCC). However, in contrast to mAb SWA11 cytoplasmic expression was observed less frequently than membranous expression in AC. In SCC, both cytoplasmic and membranous expression was rare. Normal lung parenchyma (i.e. alveolar surface cells) showed a distinct membranous immunoreactivity. Bronchial epithelium revealed both membranous and cytoplasmic staining of CD24.

*Correlation between SWA11 and SN3b:* As SWA11 and SN3b detect different epitopes, we evaluated the correlation of the immunohistochemical staining patterns. Of 132 NSCLC specimens with matched expression data, only 9 specimens (6.8 %) revealed a concordant CD24 expression. Of these cases, 4 cases revealed a concordant cytoplasmic staining and another 5 cases revealed a concordant membranous CD24 expression. Statistically, no significant correlation between the two mAb could be observed (cc = −0.63, *p* = 0.470; Fisher’s exact test *p* = 0.665). The correlation of cytoplasmic and membranous expression (for each antibody) was as follows: cc = 0.475 (*p* < 0.05) for SWA11 (*n* = 108) and cc = 0.140 (*p* = 0.11) for SN3b (*n* = 103).

### Survival analyses

Recent studies indicate that CD24 expression is associated with tumor progression and poorer survival rates. Therefore, we performed follow up analyses with a special emphasis on 1) the prognostic value of mAb SWA11 in dependence on subcellular staining characteristics and 2) the prognostic values of different clinicopathological parameters:

#### Prognostic value of CD24 in Kaplan Meier Analyses

**Fig 2:**
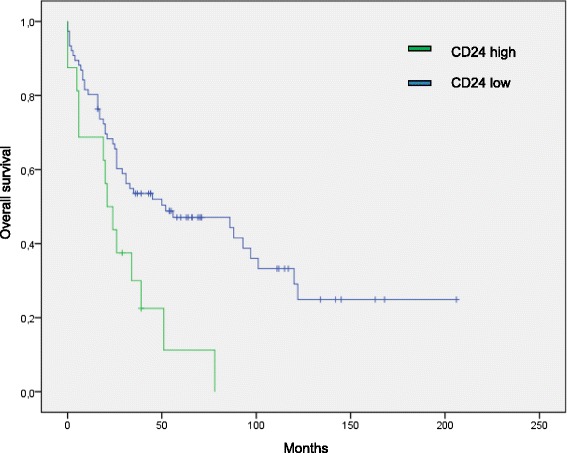
Survival analysis. Kaplan-Meier curves according to SWA11 expression. Cases with moderate to strong expression were bundled in a ‘high expression’ and cases with negative or weak expression in a ‘low expression’ group. Membranous expression of CD24 detected by SWA11 proved to be an independent marker for shorter survival times in NSCLC (*p* = 0.005)

Only membranous CD24 (SWA11) staining revealed significantly poorer survival rates (median overall survival 21 vs. 52 months; *p* = 0.005) as illustrated in Fig. 2. In contrast, cytoplasmic CD24 (SWA11) staining did not affect the survival rates (median OS 34 vs. 35 months; *p* = 0.884) (Table 3). When stratifying the cohort into SCC (*n* = 35) and AC (*n* = 102) in Kaplan Meier analyses, membranous CD24 (SWA11) expression did not affect patients’ survival, neither in SCC (*p* = 0.243) nor AC (*p* = 0.135) (Table 3), probably due to the small number of observations (Fisher exact test: *p* > 0.05). After stratification for AC subtypes, membranous CD24 expression (SWA11) showed a tendency towards an association with poorer survival in acinar subtype AC, but failed significance (*p* = 0.328).

**Table 3:** Univariate survival analysis

SWA11	No. of cases	Mean survival time	Median survival time	p-value
		(months +/− s.e.)	(months +/− s.e.)	
Mem CD24				
Negative	76	84.833 +/− 10.395	52.000 +/− 27.030	0.005
Positive	16	27.925 +/− 6.379	21.000 +/− 4.000	
Cyto CD24				
Negative	66	75.209 +/− 10.577	35.000 +/− 12.422	0.884
Positive	26	60.540 +/− 11.551	34.000 +/− 12.196	
Total CD24				
Negative	64	76.972 +/− 10.841	35.000 +/− 13.726	0.633
Positive	28	57.535 +/− 10.895	34.000 +/− 9.303	
SCC				
Mem CD24 negative	16	52.063 +/− 14.668	16.000 +/− 16.000	0.243
Mem CD24 positive	7	21.571 +/− 7.201	24.000 +/− 23.568	
AC				
Mem CD24 negative	59	88.953 +/− 11.631	56.000 +/− 22.885	0.135
Mem CD24 positive	8	39.167 +/− 11.674	21.000 +/− 8.485	
pN0	31	103.641 +/− 14.940	93.000 +/− 28.224	0.012
pN1+	30	54.911 +/− 10.646	26.000 +/− 0.983	

CD24 immunoreactivity using the mAb SN3b was not associated with patients’ survival: neither the membranous staining pattern (*p* = 0.9), nor the cytoplasmic staining pattern (*p* = 0.924) revealed any significant effect on the overall survival.

#### Prognostic values of the clinicopathological parameters

To further verify the prognostic values of different clinicopathological parameters, Cox regression analyses were conducted. As expected, positive nodal status (pN > 0) (*p* = 0.003) and disease stage (pT) (*p* = 0.006) were associated with poorer survival rates in univariate analyses (Table 4). Also, membranous CD24 (SWA11) positivity (*p* = 0.007) and histological tumour type (*p* < 0.001) showed a correlation with poorer survival rates. Subjecting the first three criteria to multivariate analyses, only membranous CD24 (SWA11) positivity (*p* = 0.014) and positive nodal status (*p* = 0.027), but not disease stage (*p* = 0.185) maintained independent factors for poorer overall survival (Table 5). In an extended multivariate Cox regression model with inclusion of tumour histology, membranous CD24 expression failed significance, but still showed a trend towards an association with shortened survival times (*p* = 0.094). CD24 expression using the mAb SN3b revealed no association with survival characteristics, neither for total (membranous or cytoplasmic) expression, nor for membranous or cytoplasmic expression alone (all *p* > 0.05, data not shown).

**Table 4:** Univariate survival analysis according to the Cox regression model (mAb SWA11)

	Beta	HR (hazard ratio)	95 % CI of HR	P-value
SWA11 mem all	0.856	2.353	1.268–4.364	0.007
pN	0.963	2.620	1.389–4.943	0.003
pT	0.844	2.325	1.279–4.224	0.006
Tumour type	0.975	2.651	1.999–3.517	0.000

**Table 5:** Multivariate survival analysis according to the Cox regression model (mAb SWA11)

	Beta	HR (hazard ratio)	95 % CI of HR	P-value
SWA11 mem all	0.944	2.571	1.211–5.458	0.014
pN	0.737	2.091	1.087–4.021	0.027
pT	0.587	1.799	0.755–4.283	0.185

## Discussion

In the present study, we have analyzed immunohistochemical staining characteristics and the prognostic value of CD24 expression in NSCLC with a special emphasis on the comparison of the CD24 antibodies SWA11 and SN3b. The most important result of our study is that the prognostic relevance of CD24 is critically dependent on the careful consideration of sub-cellular compartments and the epitope specificity of the antibody used.

Overall, about one third of the NSCLC cohort revealed a significant CD24 expression (either cytoplasmic or membranous). These results are in line with the findings of other studies. In another NSCLC cohort, CD24 (SN3b) expression was found in 33 % of the samples (87 of 267 cases) [2]. Consistent with those results, we have found similar rates of high CD24 expression levels (35 % of the cases) for SWA11. Originally, we would have expected lower rates than those found by Lee et al, as they used the antibody SN3b, that also recognizes yet unidentified other glycoproteins next to CD24. Furthermore, they used whole mount sections instead of tissue microarrays. A possible explanation for rather equal detection rates would be the fact that it has been demonstrated that the epitope recognized by SN3b is indeed present in CD24, but is not found in all glycoforms of CD24 [14]. In contrast to the commonly used mAb SN3b, mAb SWA11 binds to the protein core of CD24 and does not depict other glycan moieties next to CD24. The protein core of CD24 is linear, consisting of the amino acid sequence leucine-proline-alanine (LAP) next to a glycosyl-phosphatidylinositol anchor [15].

CD24 expression has been associated with disease progression and cancer-related death in the majority of malignant tumors [2, 3, 16, 17], although a caveat to these data is that most of these studies are based on the supposedly less specific CD24 clone SN3b. Lee et al demonstrated a significant association between CD24-high expression (SN3b) and shorter patient survival times. Furthermore, Lee and colleagues and ourselves in former studies referred the results to cytoplasmic CD24 expression [2, 3].

In invasive ovarian carcinoma, patients carrying tumors with cytoplasmic CD24 expression showed a significantly shorter mean survival time of 37 months versus patients with tumors without cytoplasmic expression of CD24 (98 months) [16]. Also in NSCLC, expression of CD24 has been claimed to be an independent prognostic marker of shorter patient survival times, especially in AC [3].

Recently, CD24 expression has been addressed as a putative stem cell marker in NSCLC [10]. In that context particular attention has to be paid to the co-expression of CD44. Sterlacci et al. demonstrated that the phenotype CD24−/CD44+ did not show a significant difference in overall survival for the entire NSCLC cohort when compared with the CD24+/CD44 −population. However, when stratified according to histology, AC displaying the putative cancer stem cell (CSC) signature CD24−/CD44+ had a significantly shorter overall survival than CD24+/CD44− AC. However, these findings could not be ascertained as an independent factor, when calculated by multivariable analysis [10]. Since the overwhelming evidence of the pro-tumorigenic properties of CD24 is independent of the CD24low/CD44-high stem cell definition, we focused on CD24 expression alone and did not include CD44 expression data in the present study.

Our results are only partly consistent with the published data. We were able to demonstrate that membranous staining pattern (using the mAb SWA11) was associated with a poorer overall survival and we revealed an increased risk of lymphonodular spread in the subgroup of CD24-high tumors, being in accordance with the published data. Nonetheless, our results are also partially conflicting with previous immunohistochemical data, as we could not confirm a significant correlation with patient survival for cytoplasmic expression of CD24, neither for SWA11 nor for SN3b.

The underlying biological mechanisms of CD24 promoted tumor progression are still incompletely characterized, although a growing number of studies have contributed to our comprehension [5–7, 17–20]. Expression of CD24 may provide an enhanced capability of tumor cells to adhere to activated endothelial cells mediated by its P-selectin binding site [5] or alter cellular signaling [21]. Aigner and colleagues showed that CD24 functions as a ligand for P-Selectin under physiological flow conditions, using a plate flow chamber assay. In their study, CD24 proved to be necessary for mediation of rolling on P-Selectin, as low expression levels or cleavage of CD24 resulted in inhibition of attachment and rolling, in a breast carcinoma cell line [6]. The precise mechanisms of ligand binding have still to be elucidated. In particular, CD24 does not contain the sulfated tyrosine residue of the P-Selectin glycoprotein ligand 1 (PSGL-1), i.e. another P-Selectin ligand [22]. Another mechanism of CD24 binding focuses on the observed association of CD24 with the sulfate-containing epitope HNK-1 which is also recognized by P-Selectin. This observation may lead to the assumption that HNK-1 mediates CD24 binding [7]. Enhanced disease progression as a result of metastatic spread with poorer survival rates may therefore be reasonable [6, 7]. As known, cells of hematogenously metastasizing tumours attach to platelets in the bloodstream [23–25]. Activated platelets express P-Selectin. Therefore, CD24-positive cells probably attach to activated platelets, containing P-Selectin on their surface, at the point, when the primary tumour invades into the vascular system [6]. Moreover, CD24-mediated tumor propagation has also been associated with an increase of local invasiveness: CD24 mediated invasion of cancer cells has been hypothesized as a result of increased contractile forces as indicated by the findings of A125 human lung cancer cells with different CD24 expression levels using CD24-high and CD24-low transfectants in three-dimensional extracellular matrix (ECM) invasion assays [19]. The percentage of invasive cells and their invasion depth was increased in CD24-high cells compared with CD24-low cells. Conversely, knockdown of CD24 and of the ß1-integrin subunit in CD24-high cells decreased their invasiveness, indicating that the increased invasiveness is CD24- and ß1-integrin subunit-dependent [19]. Interestingly, besides acting as a ligand for P-Selectin, recently it has been proven that CD24 expression also indirectly stimulates cell adhesion to fibronectin, collagens 1 and 4, and laminin through activation of *α*3*β*1 and *α*4*β*1 integrin activity. Sleeman and colleagues have reported that *β*1 integrins colocalize focally with CD24. This suggests a direct interaction between CD24 and *β*1-containing integrins. In their study they show that CD24 interacts with c-scr, leading to stabilization of the kinase-active form of c-scr, which is necessary for sufficient activation of integrin adhesion to extracellular matrix components such as fibronectin. Thus, CD24 mediates cell adhesion in a P-Selectin dependent and a P-Selectin independent manner [26]. Next to its influence on cell adhesion, metastasis and on invasion, CD24 also serves as a mediator of proliferation. Apparently, a depletion of CD24 by siRNA leads to a significant decrease of cell numbers in several cell lines as well as to a reduction of their clonogenicity [17]. These experimental results provide a functionally well compatible explanation for the observed clinicopathological correlation of CD24 expression and poorer overall survival resp. increased occurrence of lymphonodular spread in our study.

Notably, CD24 may also provide a promising target for individualized therapy strategies beyond the scope of prediction. For example, CD24 specific antibodies have been applied in the treatment of the transplantation associated B-cell proliferative syndrome [18]. Moreover, mAb SWA11 has recently been shown to have a beneficial effect on anti-cancer treatment when used as addition to gemcitabine treatment in an A549 lung cancer model [20]. Pretreatment with mAb SWA11 led to a significant retardation of carcinoma growth compared to monotherapy with gemcitabine, which was attributed to faster internalisation of tumour antigen-bound therapeutic antibodies and alterations in the intratumoural cytokine milieu. Increased levels of intratumoural chemoattractants such as CXCL9/MIG and CCL2/MCP-1 were observed, in accordance to a heightened infiltration of xenografts by macrophages, possibly gained through the involvement of the antibody-dependent cell-mediated cytotoxicity. [20]

Some parts of our results, however, are conflicting with previous data, as we could not reproduce a significant correlation with patient survival for cytoplasmic expression of CD24 for SWA11. Vice versa, we revealed that only the membranous staining pattern was indicative for poorer overall survival (*p* = 0.007). As a minimal discordance to findings of former NSCLC studies [2, 3], our SWA11 based study could not confirm an especially relevant prognostic value of CD24 for pulmonary adenocarcinomas. Still, this study demonstrates a small trend towards a subtype-dependent prognostic relevance of membranous CD24 expression. Larger cohorts will be necessary for a more substantial statistical power concerning its prognostic relevance for AC.

## Conclusions

In summary, our data provides further evidence for CD24 as a functionally relevant biomarker with prognostic significance in NSCLC. Methodically, our results underline the necessity to choose a specific antibody and to carefully consider subcellular staining differences of CD24 for robust prognostic conclusions. The use of mAb SWA11 should be favoured for a more specific detection of the cell surface protein CD24 as it allows a good visual distinction between membranous and cytoplasmic staining. A distinction between strongly and less strongly stained cells may be challenging in the light of laboratory-specific variations as well as inevitable interobserver variability. Further studies should clarify, if an adjuvant therapeutic use of CD24 antibodies may have an additive value in cancer treatment.

## Abbreviations

NSCLC: Non-small cell lung cancer
GPI: Glycosyl-phosphatidylinositol
PSA: Prostate-specific antigen
mAb: Monoclonal antibody
TMA: Tissue microarray
HE: Haematoxylin-eosin
AC: Adenocarcinomas
SC: Squamous cell carcinomas
LAP: Leucine-proline-alanine
PSGL-1: P-Selectin glycoprotein ligand 1
ECM: Extracellular matrix
